# Application of the intelligent interactive system to promote the full course care of very low birth weight infants

**DOI:** 10.3389/fped.2025.1516969

**Published:** 2025-02-28

**Authors:** Namei Xie, Ruming Ye, Yuying Chen, Ying Lin, Xianghui Huang, Deyi Zhuang

**Affiliations:** ^1^ Nursing Department, Xiamen Children's Hospital, Xiamen, China; ^2^ Nursing Department, Jinjiang Municipal Hospital, Fujian, China; ^3^ Fujian Key Laboratory of Neonatal Diseases, Xiamen Children's Hospital, Xiamen, China; ^4^ Xiamen Neonatal Quality Control Center, Xiamen, China

**Keywords:** very low birth weight infant, full course care, intelligent interactive system, intelligent nursing, preterm

## Abstract

**Aim:**

To explore the effects of full course care with an intelligent interactive system on the growth, neurodevelopment and follow up adherence of very low birth weight infants (VLBW), as well as parental satisfaction.

**Methods:**

The preterm infants admitted between January 2022 and December 2022 were designated as the experimental group, while those admitted from January 2021 to December 2021 constituted the control group. The experimental group received a full course of care through an intelligent interactive system, whereas the control group received inpatient and follow-up care from primary and clinic nurses, respectively. We compared the two groups' parental satisfaction at discharge, infants' anthropometric assessments, follow-up adherence, and infant's neurodevelopment.

**Results:**

From January 1, 2021, to December 31, 2022, 171 VLBWI and their parents were enrolled at a tertiary hospital, with 89 infants in the experimental group and 82 in the control group. Parental satisfaction in the experimental group was significantly higher than in the control group (*P* < 0.05). At the corrected ages of 1, 3, and 6 months, the height and weight of the preterm infants in the experimental group were also superior to those of the control group (*P* < 0.05). Additionally, the follow-up adherence rate of the experimental group exceeded that of the control group at the corrected ages of 3 and 6 months (*P* < 0.05). Furthermore, at the corrected age of 6 months, the scores for fine motor skills, adaptability, and social interaction of infants in the experimental group were higher than those in the control group (*P* < 0.05).

**Conclusion:**

An intelligent interactive system can facilitate comprehensive management of VLBWI by integrating inpatient care and follow-up. This approach has significant clinical value as it promotes the physical and cognitive development of infants while enhancing parent satisfaction and adherence.

## Impact

An intelligent interactive system demonstrates substantial clinical importance by enabling comprehensive management of VLBWI, achieved through the seamless integration of inpatient care and follow-up. This approach not only fosters growth, neurodevelopment, and follow-up adherence of VLBWI, but also elevates levels of parental satisfaction.

## 1 Background

With advancements in neonatal and perinatal medicine and monitoring technology, the survival rate of VLBWI, defined as those with a birth weight of less than 1,500 grams, has significantly increased. However, due to their low birth weight, low gestational age, and immature bodily systems, these infants often experience growth restrictions, neurodevelopmental delays, and other serious complications that can negatively impact their quality of life (1, 2). Additionally, the needs of VLBWI parents vary at different stages, and if these needs are not addressed, it can affect their satisfaction with healthcare providers and the overall quality of care for their infants (3, 4). The Action Promotion Plan for Healthy Children (2021–2025) also emphasizes the importance of implementing special, ongoing, information-based, and standardized management for premature infants (5). Thus, the primary focus of clinical management is to provide comprehensive, continuous, and effective health management for VLBWI to ensure their normal development. The full-course care for VLBWI refers to the comprehensive management of these infants from birth until discharge, including the hospitalization and transition periods during discharge, follow-up care, and home management. This model of care aims to ensure continuity and coordination of services to optimize health outcomes for VLBWIs. Full-course care requires collaboration among multidisciplinary teams to provide family-centered care that is tailored to meet each infant's unique needs.

Currently, the standardized management of VLBWI primarily emphasizes inpatient care. However, no uniform standards regarding service content, format, and system have been established for transitional and post-discharge care. Additionally, specialized leadership and intelligent information support tools for inpatient and post-hospital management are lacking. This makes it challenging to track the health status and prognosis of VLBWI dynamically. The continuity and individualized assessment of VLBWI is often inadequate, which creates difficulties in providing comprehensive care (6–9).

We developed an intelligent interaction system to provide comprehensive care for VLBWI. This system incorporates scene-based knowledge expression and integrates information interactions within our hospital (10, 11). Leveraging big data and artificial intelligence algorithms enables interaction with medical and nursing information, helping healthcare professionals deliver continuous and efficient health management for VLBWI. In addition, we have integrated the concept of case management into our approach and established a VLBWI case management team led by a specialized nurse (12–14). This nurse facilitates communication with families and coordinates available resources to address the diverse needs of VLBWI and their families at various stages of care. Through this project, we aimed to enhance the growth and neurological development and follow-up adherence of VLBWI, as well as improve their parents’ satisfaction.

## 2 Methods

### 2.1 Study design

Non-synchronous controlled experiment.

### 2.2 Participants and enrollment

This study employs a convenience sampling method. The VLBWI and their parents hospitalized from January to December 2022 were the experimental group, and the VLBWI and their parents hospitalized from January to December 2021 were the control group. Inclusion criteria: ①1,000 g < birth weight < 1,500 g, gestational age < 34 weeks; ② Apgar score at birth ≥4 points; ③ No chromosome abnormality or other congenital malformations; ④ Informed consent was obtained from the VLBWI parents, and there were no cognitive or communication disorders. Exclusion criteria: ① The infant or family member cannot participate in the whole care; ② The infants are complicated with severe complications or severe sequelae, such as severe neonatal hypoxic-ischemic encephalopathy and intraventricular hemorrhage of grade IV. This research protocol has been approved by the Scientific and Ethical Committee of Xiamen Children's Hospital [(2021) No.17].

### 2.3 Full course care for VLBWI with an intelligent interactive system

#### 2.3.1 Team formation and responsibilities

The project team is composed of a management team and an implementation team. The management team includes one head nurse and one department director from the neonatal department to oversee the project implementation process. The implementation team consists of six nursing teams, each with seven members, led by a neonatal specialty nurse and nine pediatric doctors and staffs from the Information Department. The nursing teams and the pediatric doctors manage the care of VLBWI from hospitalization through to post-discharge follow-up using an intelligent interactive system, with support from the Information Department staff for system maintenance.

Qualification of neonatal specialized nurse: ① Bachelor degree or higher; ② A minimum of 5 years of experience working in a Neonatal Intensive Care Unit (NICU).; ③ Intermediate certificated or higher; ④ Proficiency in various NICU nursing skills and a strong foundation of professional theoretical knowledge.; ⑤ Completion of systematic specialized training, including neonatal neuropsychological behavior development evaluation, nutrition evaluation, and early family intervention.;⑥Excellent communication skills and strong organizational coordination abilities.

#### 2.3.2 Construction of the intelligent interactive system

(1) **System Construction Concept:** This system is designed to meet the needs of infants and their families by facilitating multi-dimensional interactions among key stakeholders, including doctors, nurses, infants, and caregivers. It also supports the continuum of care across inpatient nursing, outpatient follow-up, and home care. Utilizing intelligent technology and interactive methods, individualized care plans are generated through accurate evaluations at each stage. This approach aims to optimize comprehensive, efficient, and continuous disease-specific management for VLBWI.
(2) System construction methods: ① Utilize a Browser-Server (B/S) architecture, combined with Java, for system and module development.; ② Data Interaction: Facilitate data interaction by integrating with various systems, including the Hospital Information System (HIS), Laboratory Information System (LIS), Electronic Medical Records System (EMRS), Picture Archiving and Communication System (PACS), and others. This integration allows for information input by doctors, nurses, and caregivers, enabling interactive information sharing. ③ Decision support: We utilize knowledge graphs to enable the automatic analysis and processing of data, facilitating interactions between the system and the user. This approach helps users quickly and accurately obtain and understand information. In the first stage, our research group collaborated with computer engineers to construct a knowledge graph for the full-course care of VLBWI. This graph was developed based on 512 real cases of VLBWI analyzed by our team. The multidisciplinary team created personalized care plans that served as the VLBWI care case base. This case base was derived from actual clinical cases, integrating evidence from standardized clinical guidelines, systematic reviews, and the practical experience of seasoned caregivers and multidisciplinary experts. Next, we used knowledge extraction technology to extract entities and their inter-relationships from the VLBWI care case base, resulting in “entity-relationship-entity” triplet data stored in a database to complete the knowledge graph construction. In the second stage, we integrated the established knowledge graph of VLBWI care into a recommendation model using graph embedding techniques. This model consists of a graph embedding module and a recommendation module. The graph embedding module learns the features of the knowledge graph. In contrast, the recommendation module interacts with these features and the items within the recommendation system through intelligent algorithms, ultimately producing personalized recommendations for care plans. ④Develop assessment content and interventions. Based on the nursing knowledge base for VLBWI established in the initial stages, nurses who have undergone evidence-based training have updated this knowledge with the assistance of relevant guidelines, consensus statements, and evidence summaries. The methods used for updating included expert consultations, literature reviews, and content analysis. Finally, a software engineer inputs the updated content into the system, ensuring the knowledge base is regularly refreshed.
(3) Ports. The system has two ports: the administrative interface and the user interface. The administrative interface is used by team members and installed on the hospital's computer. The user interface is installed on mobile phones as an app that VLBWI parents use.
(4) Module and function. The system includes four modules: hospitalization management, follow-up management, home management, and auxiliary decision-making. Each module encompasses specific functions. ① Hospitalization management involved three key functions: “health record”, “clinical database”, and “evaluation of the discharge transition period”. These functions were utilized to establish the VLBWI cohort, manage the clinical information of infants, and assess the discharge transition period. ②Follow-up management encompasses five key functions: “Follow-up Plan”, “Temporary Follow-up”, “Development Evaluation”, “Comprehensive Query”, and “Health Education”. These functions create follow-up plans, send reminders, facilitate information queries, assess development, and provide health education. The Development Evaluation module focuses on two main areas: monitoring physical growth and evaluating neuropsychological and behavioral development. The neuropsychological and behavioral development monitoring includes various assessments such as the Neuromotor Assessment (20 items), the Test of Infant Motor Performance, the Ages and Stages Questionnaires, Third Edition (ASQ-3), the Ages and Stages Questions: Social-Emotional (ASQ: SE), the Griffiths Mental Development Scale, and the Gesell Developmental Evaluation Scale. This module can automatically recommend evaluation items based on the VLBWI age and existing high-risk factors. Additionally, it generates intervention plans based on the evaluation results. ③ Home management encompasses four key functions: A. Risk Early Warning: Risk assessment rules are established based on the infant's age, symptoms, and signs related to common complications. Family members can sequentially enter relevant information as prompted by the system. The system then automatically determines whether the infant is at risk. The evaluation results are sent to the family members and medical staff simultaneously to aid decision-making.B. Development Monitoring: This function combines early development milestones with growth development curves to automatically evaluate whether the infant is at risk for developmental issues based on the input provided. Family members can monitor the infant's growth and development at home.C. Online Consultation: This module allows families to initiate conversations or leave messages for the care team leader, who will respond within 24 h. If necessary, the team leader can also contact the doctor.D. Appointment for Doctor Visits Parents can conveniently schedule doctor appointments through the app. ④ Assisted decision support: The system automatically assessed whether there were clinical issues that needed to be addressed or high-risk factors for development in infants based on the data entered at each stage. It intelligently generated individual plans for diagnosis, treatment, nursing, and home-based medical care as part of its decision-making process.
(5) System verify: Before training the algorithm, we thoroughly cleaned and preprocessed the original data. This included removing duplicate records, filling in missing values, and correcting any errors to minimize the impact of inaccurate data on the algorithm's performance. Next, we analyzed feature importance to identify the most critical variables for creating personalized nursing plans. We continuously optimized the algorithm model to enhance its robustness and generalization capabilities. Additionally, we divided the data from 512 cases of VLBWI into training and validation sets using cross-validation, allowing us to preliminarily train and test the algorithm. In practical applications, we established a real-time monitoring system to continuously track and evaluate the nursing plans generated by the algorithm. If any abnormalities or deviations are detected, a feedback mechanism is activated immediately to fine-tune or retrain the algorithm.

### 2.4 Interventions

#### 2.4.1 Control group

During the hospitalization, the primary nurses and attending doctors provided care for VLBWI. Upon discharge, the primary nurses offered guidance on managing the child's condition and educated the parents about health care practices. They instructed the parents on home care methods for VLBWI, including bathing, feeding, and monitoring the child's health. Additionally, the nurses informed the parents about the time and location of the outpatient follow-up visit to ensure they could attend. After discharge, the nurses at the follow-up center conducted visits and provided a paper record to track the child's progress.

#### 2.4.2 Experimental group

The experimental group received full course management facilitated by an intelligent interactive system overseen by a specialty nurse. Specialty nurses play a crucial role in evaluating outcomes at various stages, coordinating resources to address the individual needs of infants and their families, and offering proactive, continuous, and seamless health management services.

(1) **Hospitalization**: After the infant was admitted, the specialty nurse interviewed the family members, explained the related procedures, and provided information about the overall care plan. The informed consent form was then signed. An intelligent interactive system established a queue for VLBWI. The system automatically captured and imported the infant's basic medical history, treatment records, examination results, and nursing notes into a comprehensive clinical database. This “health record” allowed for dynamic observation of the infant's disease progression and the effectiveness of the medical care provided. The nursing team was guided in creating an individualized care plan, coordinating and organizing medical activities with the attending physician, and facilitating communication and information sharing among all three parties involved in the care.
(2) **Discharge transition**: This period encompasses the two weeks leading up to the discharge of a VLBWI. During this time, the infant's physician conducts an evaluation to prepare for discharge. A specialty nurse assesses the family members' knowledge, skills, and needs related to home care through a “Transitional Period Assessment” module. Based on the evaluation results, the nurse develops a training plan for the family, guides the nursing team in implementing the training, and evaluates its effectiveness. Family members who complete the training are allowed to participate in the infant's bedside care under the nursing team's supervision.Before discharge, the specialty nurse and the doctor demonstrate the treatment and care outcomes for the infant using the “Health File—Patient View” module. They emphasize the importance of follow-up care and collaboratively create an individualized follow-up plan with the family using the “Follow-Up Plan” module. Based on the entered data, the system automatically generates follow-up cycles and items tailored to the infant. The specialty nurse guides the family on using the mobile app. Discharge criteria: After the children were evaluated by the physicians with the titles of doctor in charge and above, they met the discharge criteria in the Specifications for Health Care of Premature Infants (15), that is, they “weighed ≥ 2,000 g, had stable vital signs, could be fed with adequate oral feeding, had sustained weight growth, could maintain normal body temperature at room temperature, and could be discharged after the disease was cured or family sequential treatment”.
(3) **Post-discharge follow-up and home care**: The care team leader is responsible for overseeing the post-discharge follow-up of VLBWI and managing and maintaining the intelligent interactive system. Following the established follow-up schedule (15) (Table 1), the tasks are communicated to the specialty nurse and the infant's family three days in advance through the “Temporary Follow-up” module. During the follow-up visit, the specialty nurse can access the infant's previous medical information and home nursing information through the “comprehensive query” function and conduct targeted follow-up evaluation with the help of the “development evaluation” module to accurately evaluate the development of the infants at each stage. The “Decision Support” module can automatically generate a development evaluation report based on the results entered by the specialty nurse. It also provides early intervention and home care recommendations for infant. Based on these evaluation results, the specialty nurse has the ability to add or remove suggested care measures. Additionally, specialty nurses can share tailored and general health education information with children's families through the “Health Education” module. The “Home Management” module assists families in monitoring infant's development, offers early warning signals for potential risks, and addresses common complications. It also provides online consultation and appointment scheduling options for families.

**Table 1:** Time and frequency of follow-up (15).

Time of follow-up	Frequency of follow-up
Within Corrected age of one month	Once every two week
Corrected age of 1st month to 6th month	Once every month
Corrected age of 7th months to 12th month	Once every two months
Corrected age of 13th months to 24th month	Once every three months
Corrected age above 24th month	Once every six months

### 2.5 Tools

#### 2.5.1 Growth and development assessment

Z-score was used to evaluate the development of height and weight. The Z score was assessed using the physical growth indicators scale for normal children aged 0–2, released by WHO in 2006, as the reference standard (16). Z = (individual measured value−reference mean)/standard deviation, Z score < −2 was not up to catch-up growth level, Z score ≥ −2 was catch-up growth level, and Z score > 0 was medium to upper level.

#### 2.5.2 Neuropsychological development assessment

It was measured with the Gesell Development Diagnostic Scale (17), including gross motor, fine movement, adaptive behavior, language, and personal-social behavior. Developmental quotient (DQ) was used to represent the developmental level of infants and young children. DQ = developmental age/actual age × 100. A score of DQ greater than 85 indicated normal neurodevelopment and a score from 75 to 85 indicated a critical level of neurodevelopmental retardation. A score lower than 75 indicated neurodevelopmental retardation.

#### 2.5.3 Parental satisfaction

The NICU Parental Satisfaction Scale, translated by Zhuang (18), was utilized to assess parental satisfaction regarding their infant's care. This scale encompasses five dimensions of medical care: professional attitude, parents' participation, information, and organization, consisting of a total of 30 items. Using a six-point Likert scoring system, responses are rated from one to six, with one indicating “very dissatisfied” and six indicating “very satisfied”. A higher score reflects a greater degree of satisfaction. The Cronbach's α coefficient for the overall scale was found to be 0.90.

#### 2.5.4 Follow-up adherence

The follow-up rate at each stage is calculated as the number of complete participants divided by the total number of participants at that stage, multiplied by 100%.

#### 2.5.5 System usability scale

System Usability Scale (SUS) is a standardized questionnaire widely used to evaluate users' perceived usability with good reliability (Cronbach's α = 0.91) (19). It is widely used to assess the usability of websites, software, mobile devices, and medical systems, and only a small sample size (*N* < 14) is needed to obtain a reliable experimental result. The measured scores can comprehensively show the usability level. The questionnaire adopts the Likert 5-scale scoring method. Users can choose five options from “strongly agree” to “strongly disagree” according to their feelings; the score ranges from 0 to 100 (19). The higher the score, the higher the user satisfaction, so the system has high usability. A score greater than 70 indicates high system applicability, and a lower than 70 indicates low system applicability. The score can effectively reflect the user's satisfaction with system services and products.

### 2.6 Data collection methods

At the time of discharge, the satisfaction of parents from both groups was evaluated. The follow-up adherence and physical development of the children were assessed at corrected gestational ages of 1, 3, and 6 months. Additionally, at the corrected gestational age of six months, the neurodevelopmental status of the two groups of children was evaluated. For the control group, follow-up data were collected using the VLBWI follow-up record card after discharge, while the intelligent interactive system was utilized to collect and manage all care information for the experimental group.

### 2.7 Quality control

In this study, the follow-up adherence of VLBWI was improved through three key measures: ① Health education was provided before discharge to emphasize the importance and necessity of timely follow-up for VLBWI, which increased family members' awareness of the need for prompt follow-up. ② Utilizing the “Temporary Follow-up” of an intelligent interactive system, a reminder message was automatically sent to family members three days before their scheduled visit, encouraging them to attend on time. ③ Timely communication and attention to feedback were prioritized, with specialty nurses actively responding to family members' inquiries within 24 h.

Additionally, the study included unified training and assessment for the intervention measures and data collection tools used for VLBWI in both groups prior to the intervention. Data entry was performed by two individuals and reviewed by a designated nursing team leader to ensure the consistency of the intervention implementation and the standardization and accuracy of the data collected.

### 2.8 Statistical methods

SPSS version 20.0 was utilized to create a database for double data entry and to conduct statistical analysis. Continuous variables that followed a normal distribution were reported as mean ± standard deviation (x¯ ± s), while categorical variables were presented as frequencies and proportions. An independent sample t-test was applied for normally distributed data, a rank-sum test was used for non-normally distributed continuous data, and non-parametric tests were employed for categorical data. All statistical tests were conducted bilaterally, and a difference was considered statistically significant if the *P*-value was less than 0.05.

## 3 Results

### 3.1 General information

Ninety-five cases were initially included in the experimental group, while 90 cases were included in the control group. In the experimental group, three infants were transferred for follow-up, and another three were lost to follow-up by the time they reached a corrected age of six months. Three infants were transferred for follow-up in the control group, and five were lost to follow-up after three months corrected age. Consequently, the final numbers included in the analysis were 89 infants in the experimental group and 82 infants in the control group. There were no significant differences between the two groups in terms of gender, birth weight, gestational weeks, birth mode, Apgar score, complications, hospital stays, or demographic characteristics of the VLBWI parents (*P* > 0.05), as shown in Table 2.

**Table 2:** General information of VLBWI and their parents in two groups.

General Information	control group (*n* = 82)	Experimental group (*n* = 89)	*T/χ* value	*P* value
VLBWI Gender [Cases (%)]				
Girl	37 (45.2)	41 (46.9)	0.0	0.87
Boy	45 (54.8)	47 (53.1)		
Gestational weeks (weeks, $\bar{x}\pm s$)	28.2 ± 0.9	28.1 ± 1.2	0.4	0.69
Birth weight (g, $\bar{x}\pm s$)	1 249.1 ± 56.1	1 256.1 ± 47.1	−0.7	0.52
Birth mode [Cases (%)]				
Cesarean section	10 (11.9)	5 (6.1)	0.9	0.33
Vaginal	72 (88.1)	84 (93.9)		
Apgar score at birth ($\bar{x}\pm s$)	7.0 ± 1.0	7.2 ± 1.1	−0.8	0.45
Neonatal respiratory distress syndrome[cases (%)]	41 (50.0)	45 (51.0)	0.010	0.92
Neonatal necrotizing enterocolitis [Cases (%)]	6 (7.1)	4 (4.1)	0.4	0.52
Bronchopulmonary dysplasia [Cases (%)]	19 (23.8)	24 (26.5)	1.1	0.31
Hospital stay (days, $\bar{x}\pm s$)	74.9 ± 6.2	74.8 ± 6.5	0.0	0.99
Maternal Age (years,$\bar{x}\pm s$)	27.8 ± 2.6	28.6 ± 2.5	−1.8	0.08
Maternal Education [case (%)]				
High school and below	33 (40.5)	38 (42.9)	0.1	0.82
University and above	49 (59.5)	51 (57.1)		
Paternal age (years, $\bar{x}\pm s$)	28.0 ± 1.5	28.9 ± 2.9	−1.8	0.08
Paternal Education [case (%)]				
High school and below	37 (45.2)	43 (49.0)	0.1	0.72
University and above	45 (54.8)	46 (51.0)		

### 3.2 Growth and development

Significant differences in the z-value distribution of height and weight were observed between the two groups of VLBWI following at the correct age of one, three, and six months (*P* < 0.05). Besides, the number of children whose height/weight developed to the medium upper level or at the catch-up growth level in the control group was less than that in the experimental group at all time points, while the number of children who did not reach the catch-up growth level was more than that in the experimental group at all time points. See Tables 3, 4 for details.

**Table 3:** Comparison of the Z-value distribution of weight at each corrected month between two groups.

Corrected month		Control group (*n* = 82)	Experimental group (*n* = 89)	*χ* value	*P* value
One month for correction	<−2	25 (31.0)	9 (10.2)	7.4	0.02
	−2–0	49 (59.5)	60 (67.4)		
	>0	8 (9.5)	20 (22.4)		
3 months for correction	<−2	15 (19.0)	0 (0.0)	7.0	0.03
	−2–0	51 (61.9)	62 (69.4)		
	>0	16 (19.1)	27 (30.6)		
6 months for correction	<−2	10 (11.9)	0 (0.0)	6.2	0.04
	−2–0	33 (40.5)	42 (46.9)		
	>0	39 (47.6)	47 (53.1)		

**Table 4:** Comparison of the Z-value distribution of height at each corrected month between two groups.

Corrected month		Control group (*n* = 82)	Experimental group (*n* = 89)	*χ* value	*P* value
One month for correction	<−2	23 (28.6)	7 (8.2)	7.1	0.02
	−2–0	49 (59.5)	62 (69.4)		
	>0	10 (11.9)	20 (22.4)		
3 months for correction	<−2	20 (23.8)	4 (4.1)	7.7	0.02
	−2–0	39 (47.6)	53 (59.2)		
	>0	23 (28.6)	32 (36.7)		
6 months for correction	<−2	14 (16.7)	0 (0.0)	9.0	0.01
	−2–0	27 (33.3)	40 (44.9)		
	>0	41 (50.0)	49 (55.1)		

### 3.3 Neurodevelopmental outcomes at 6 months

When the correction age was six months, there was no significant difference in the two groups' gross motor and language ability scores of VLBWI (*P* > 0.05). However, the fine motor, adaptability, and personal social ability scores of VLBWI in the experimental group were higher than those in the control group, and the difference was statistically significant (*P* < 0.05), as shown in Table 5. At the corrected six-month age, the development levels of VLBWI were classified and compared according to the VLBWI developmental quotient. The results showed that the number of VLBWI in the experimental group at the normal and critical levels of development in the adaptive and personal-social was significantly higher than in the control group (*P* < 0.05), as shown in Table 6.

**Table 5:** Comparison of the DQ values at corrected six months between two grops ($\bar{x}\pm s$).

DQ values	control group (*n* = 82)	Experimental group (*n* = 89)	*T* value	*P* value
Gross motor	76.4 ± 1.9	76.6 ± 1.8	−0.7	0.47
Fine motion	76.2 ± 1.7	76.9 ± 1.3	−2.1	0.03
Adaptability	76.4 ± 1.9	79.1 ± 2.9	−5.4	<0.00
Language ability	76.3 ± 1.1	77.6 ± 0.9	−1.2	0.25
Personal-social	76.2 ± 2.0	78.6 ± 2.8	−4.5	<0.00

**Table 6:** Comparison of development levels at corrected six months between two groups.

Developmental level	Big sport		Fine motion		adaptability		Language ability		Personal-social	
	Control group (*n* = 82)	Experimental group (*n* = 89)	Control group (*n* = 82)	Experimental group (*n* = 89)	Control group (*n* = 82)	Experimental group (*n* = 89)	Control group (*n* = 82)	Experimental group (*n* = 89)	Control group (*n* = 82)	Experimental group (*n* = 49)
Normal	0 (0.0)	0 (0.0)	0 (0.0)	0 (0.0)	0 (0.0)	7 (8.2)	0 (0.0)	0 (0.0)	0 (0.0)	5 (6.1)
Critical level	70 (85.7)	78 (87.8)	70 (85.7)	85 (95.9)	70 (85.7)	82 (91.8)	82 (100.0)	89 (100.0)	70 (85.7)	84 (93.9)
Slow	12 (14.3)	11 (12.2)	12 (14.3)	4 (4.1)	12 (14.3)	0 (0.0)	0 (0.0)	0 (0.0)	12 (14.3)	0 (0.0)
*χ* value	0.1		2.9		10.3		—		9.9	
*P* value	0.77		0.09		<0.00		—		<0.00	

### 3.4 Parental satisfaction and follow-up adherence

At discharge, the satisfaction scores and total scores of parents in treatment care, information acquisition, professional attitude, parental participation, and organization of VLBWI in the experimental group were higher than those in the control group (*P* < 0.01). See Table 7 for the difference. In addition, within one, three, and six months corrected age, the percentages of scheduled participation of VLBWI in the control group and the experimental group were 80.95%, 59.52%, 52.38%, and 91.84%, 81.63%, and 73.47%, respectively. The follow-up adherence of VLBWI in the experimental group at three and six months corrected age was significantly higher than that in the control group (*P* < 0.05). See Table 8.

**Table 7:** Comparison of the parental satisfaction between two groups ($\bar{x}\pm s$).

Parental satisfaction	control group (*n* = 82)	Experimental group (*n* = 89)	*T* value	*P* value
Therapeutic Care	35.1 ± 2.7	41.2 ± 2.8	−10.5	<0.01
Information acquisition	19.6 ± 1.5	22.9 ± 2.4	−5.4	<0.01
Professional attitude	25.2 ± 1.9	27.8 ± 2.2	−7.8	<0.01
Parental involvement	24.9 ± 1.3	31.0 ± 1.9	−16.1	<0.01
organization	19.9 ± 1.5	24.4 ± 2.4	−10.5	<0.01
aggregate score	124.8 ± 4.7	147.3 ± 6.2	−18.4	<0.01

**Table 8:** Comparison of the follow-up adherence between two groups [cases (%)].

Follow-up adherence		Control group (*n* = 82)	Experimental group (*n* = 89)	*χ* value	*P* value
Within 1 month of correction	Disobey	16 (19.1)	7 (8.2)	2.3	0.13
	comply with	66 (80.9)	82 (91.8)		
Within 3 months of correction	Disobey	33 (40.5)	16 (18.4)	5.4	0.02
	comply with	49 (59.5)	73 (81.6)		
Within 6 months of correction	Disobey	39 (47.6)	24 (26.5)	4.4	0.04
	comply with	43 (52.4)	65 (73.5)		

### 3.5 The clinical applicability of the system

Twenty-three participants, 9 NICU neonatal physicians, 7 specialized nurse, and 7 VLBWI caregivers were invited to conduct the study's usability assessments. The total usability score measured by the ten questions of the SUS instrument presented a mean of 81.1 for the neonatal physicians' group, 82.5 for the specialized nurse' group, and 80 for the VLBWI caregivers' group. All results were classified as excellent (72.5–85), considering the adjective scale and acceptability ranges (>68).

## 4 Discussion

### 4.1 The specialized nurse-led multidisciplinary collaboration serves as the cornerstone for addressing the diverse health needs of VLBWI

The case management mode focuses on active service and one-on-one continuous follow-up, possessing the characteristics of a responsibility system and holistic management. It has been widely used in the management of chronic diseases such as hypertension and diabetes. However, there is still a lack of relevant clinical practice in applying VLBWI management in China (20–22). In this study, the characteristics of the specially-assigned person in charge of case management, active tracking, continuous medical service, and follow-up were used for reference (12–14, 23), and the whole management mode of VLBWI intelligent interaction system led by specialty nurse was constructed. This model specifies the access standards and work responsibilities of the specialty nurse and the nursing team. As the core member of the entire nursing team, the specialty nurse is responsible for the coordination, cooperation, and communication among multiple personnel, disciplines, departments, and links in the VLBWI treatment and nursing activities, leading the planning, coordination, review, and supervision of the VLBWI inpatient-follow-up-home care related nursing activities, and obtaining a relatively comprehensive grasp of the overall situation of medical care for children. A timely and effective response to the diversified health service needs of children and their families is conducive to establishing a harmonious nurse-patient relationship. This study showed that parents' satisfaction and overall satisfaction in the experimental group in five aspects of medical care, professional attitude, participation, information, and organization were significantly higher than those in the control group.

### 4.2 Implementing intelligent clinical management for VLBWI through leveraging information interactive management and auxiliary decision support

“Information interactive management” is one of the main features of the VLBWI holistic care model. The head of the nursing team can collect, store, manage and track the data of each stage from hospitalization to follow-up for a long time by virtue of the intelligent interaction system, the electronic information continuous recording and statistical inquiry functions avoid the problems of difficult inquiry of paper follow-up records, discontinuous patient information and the like, effectively link the information in hospitalization-follow-up-home care of the children, realize the interconnection and intercommunication of the whole medical care information of the VLBWI, It was conducive for specialty nurse to conduct a comprehensive internal cause analysis and judgment on the health problems of children during the follow-up process, facilitate the continuous tracking and evaluation of the health status of VLBWI after discharge, ensure the seamless connection of nursing services for children during hospitalization and after discharge, and had a significant effect on improving the follow-up adherence of VLBWI. This study showed that the follow-up adherence of the experimental group was significantly higher than that of the control group when VLBWI was corrected for three months and six months.

“decision support” is one of the characteristic functions of an intelligent interactive system. With the support of intelligent assistance, the memory of medical staff on the calculation rules of VLBWI evaluation results can be reduced, and the system can automatically determine whether a child has a clinical problem to be treated or a development high-risk factor according to the data results input at each stage of VLBWI management. Second, the system can automatically generate personalized diagnosis and treatment suggestions and care plans according to the evaluation results so that nurses can refer to the care plans and execution forms to implement the corresponding measures, reduce the workload and cognitive load of medical care, and improve the clinical management effect of VLBWI. The results of this study showed that the total care mode based on an intelligent interaction system, led by a specialty nurse, had a positive effect on the physical development of VLBWI at the correction of one-month-old, three months, and six months old, and on the neuropsychological behavioral development at the correction of six months old, indicating that this care mode could promote the physical and intellectual development of VLBWI.

## 5 Limitation

The limitations of this system come from its user interface, which is currently only available for Android devices. An installation program for iOS systems is in development. Furthermore, both the administrative and user interfaces require training for effective use. This training ensures that healthcare professionals and family members can accurately utilize the system, ultimately enhancing its efficiency and quality. It is also important to note that this study is based on a small, historically controlled sample, and the research is limited to VLBWI. In the future, we can establish a cohort of premature infants with various conditions and conduct a multicenter study to explore the clinical benefits of comprehensive care using an intelligent interactive system.

## 6 Conclusion

The development of the intelligent interactive system the VLBWI full course care mode based on an intelligent interactive system which can improve the growth, neurological development of VLBWI, parental satisfaction and follow up. In this system, nursing leader play a key role, guiding the entire process from hospitalization to transition, outpatient follow-up, and home care for VLBWI. It facilitates active monitoring and a continuous, seamless connection for health management, addressing the individual and phased needs of infants and their families. Additionally, it promotes the sharing of information throughout the entire care process among healthcare providers and families, serving as a valuable reference for establishing comprehensive care for VLBWI.

## Data Availability

The raw data supporting the conclusions of this article will be made available by the authors, without undue reservation.
